# The efficacy and safety of bilateral synchronous transcutaneous auricular vagus nerve stimulation for prolonged disorders of consciousness: a multicenter, double-blind, stratified, randomized controlled trial protocol

**DOI:** 10.3389/fneur.2024.1418937

**Published:** 2024-05-31

**Authors:** Yan Wang, Li Yang, Wei Liu, Qianhui Zhou, Meiling Huang, Leyao Zou, Zhen Feng, Yang Bai

**Affiliations:** Affiliated Rehabilitation Hospital, Jiangxi Medical College, Nanchang University, Nanchang, China

**Keywords:** transcutaneous auricular vagus nerve stimulation, coma recovery scale-revised, heart rate variability, randomized controlled trial, disorders of consciousness

## Abstract

**Background:**

Treatment of disorders of consciousness (DOC) poses a huge challenge for clinical medicine. Transcutaneous auricular vagus nerve stimulation (taVNS) is a non-invasive neuromodulation method, which shows potential in improving recovery of DOC. However, the evidence came from single-center, small-sample randomized controlled trial, which is insufficient to form a conclusion. Thereby, we propose a prospective, multicenter, double-blind, stratified, two-arm randomized controlled trial protocol to investigate the efficacy and safety of bilateral synchronous taVNS for treatment of DOC.

**Methods:**

We aim to recruit 382 patients with prolonged DOC, and divide them into an active stimulation group and a sham stimulation group. The patients in the active stimulation group will receive bilateral synchronous taVNS with a 200 μs pulse width, 20 Hz frequency, and personal adjusted intensity. The sham stimulation group will wear the same stimulator but without current output. Both groups will receive treatment for 30 min per session, twice per day, 6 days per week lasting for 4 weeks. The clinical assessment including Coma Recovery Scale-Revised (CRS-R), Full Outline of Unresponsiveness (FOUR), Glasgow Coma Scale (GCS), and Extended Glasgow Outcome Scale (GOS-E) will be conducted to evaluate its efficacy. Heart rate variability (HRV), blood pressure, and adverse events will be recorded to evaluate its safety.

**Discussion:**

These results will enable us to investigate the efficacy and safety of taVNS for DOC. This protocol will provide multicenter, large-sample, high-quality Class II evidence to support bilateral synchronous taVNS for DOC, and will advance the field of treatment options for DOC.

**Clinical trial registration:**https://www.chictr.org.cn/showproj.html?proj=221851, ChiCTR2400081978.

## Introduction

Disorders of consciousness (DOC) refer to the state in which an individual’s response to external stimuli is reduced or even non-responsive. The DOC caused by diseases such as traumatic brain injury, intracerebral hemorrhage, cerebral infarction, and cardiac arrest, which are manifested as alterations in arousal and/or awareness (1), including coma, vegetative state/unresponsive wakefulness syndrome (VS/UWS), and minimally conscious state (MCS) (1, 2). Coma is defined as a state with completely lack of arousal (eyes closed) and awareness (3). While VS/UWS is defined as preserved arousal (eyes open) but without awareness (4). MCS is defined as the minimal, reproducible, but inconsistent state of awareness (5), without (MCS-) or with (MCS+) evidence of language function (6). In recent years, with the rapid development of modern medicine, the increasingly successful treatment for severe brain injury patients has led to a continuous increase in the number of patients falling into long-term survival with DOC (7). A DOC lasting up to 28 days is termed as a prolonged DOC (pDOC) (8). It poses a huge challenge for clinical medicine, as well as a huge pressure on families and society (7).

Numerous researchers and clinicians are devoting to improve the conscious state of patients and accelerate their recovery. Medications (amantadine, midazolam, intrathecal baclofen, etc.), invasive and non-invasive brain stimulation (deep brain stimulation, spinal cord stimulation, transcranial direct current stimulation, repeated transcranial magnetic stimulation, etc.), sensory stimulation (motor-based therapy, auditory-based training, music therapy, and multi-sensory training), hyperbaric oxygen and other treatments have been used to achieve better rehabilitation goals (9). The evidence-based basis for these treatments has been continuously improved in recent years (10, 11). Vagus nerve stimulation (VNS) is a type of brain stimulation technique, which has been considered as one of the latest neuromodulation methods benefit to patients with DOC.

The first clinical application of VNS was the treatment for intractable partial seizures (12). Its clinical application was approved by the Food and Drug Administration (FDA) in 1997 (13). Currently, besides intractable partial seizures, the FDA has approved VNS for medication-resistant depression (14), episodic cluster headaches (15) and moderate-to-severe upper extremity motor impairments following chronic ischemic stroke (16).

According to the International Consensus Based Review and Recommendations for Minimum Reporting Standards in Research on Transitional VNS (Version 2020) (17), there are four currently accepted VNS methods: cervically implanted VNS (iVNS), transcutaneous cervical VNS (tcVNS), transcutaneous auricular VNS (taVNS), and percutaneous auricular VNS (paVNS). Among them, taVNS is a safe, non-invasive, and easy-to-use treatment option, compared to iVNS (18). The first use of VNS for DOC was published in 2017 with a case report (19). A VS/UWS due to cardiac arrest developed to MCS after 4 weeks of taVNS. In the same year, another case report also reported that a patient with VS/MCS developed to MCS after 4 weeks of iVNS (20). Subsequently, 5 (21), 10 (22), and 14 (23) patients with DOC were treated with VNS in 3 case series (1 iVNS and 2 taVNS). These case series indicated that VNS improved the behavioral responses (conscious state) of patients and was safe and feasible for DOC.

However, these uncontrolled case reports (19, 20) and case series (21–23) only provided weak Class IV and Class V evidence of treatment efficacy. It cannot be ruled out that the impacts are from spontaneous recovery, especially considering the acute to subacute background of the enrolled patients. Recently, we provided the highest level of evidence for the efficacy and safety of VNS for DOC with a single-center double-blind randomized controlled trial (RCT) (28 active versus 29 sham stimulation) (24). It indicated that 4 weeks’ taVNS significantly improved the Coma Recovery Scale-Revised (CRS-R) score of MCS patients, and without significant side effects. In order to further confirm and validate the efficacy and safety of taVNS, as well as to provide more comprehensive and reliable evidence, we propose here a multicenter, double-blind, stratified, two-arm RCT.

## Methods and analysis

### Study design

**Figure 1:**
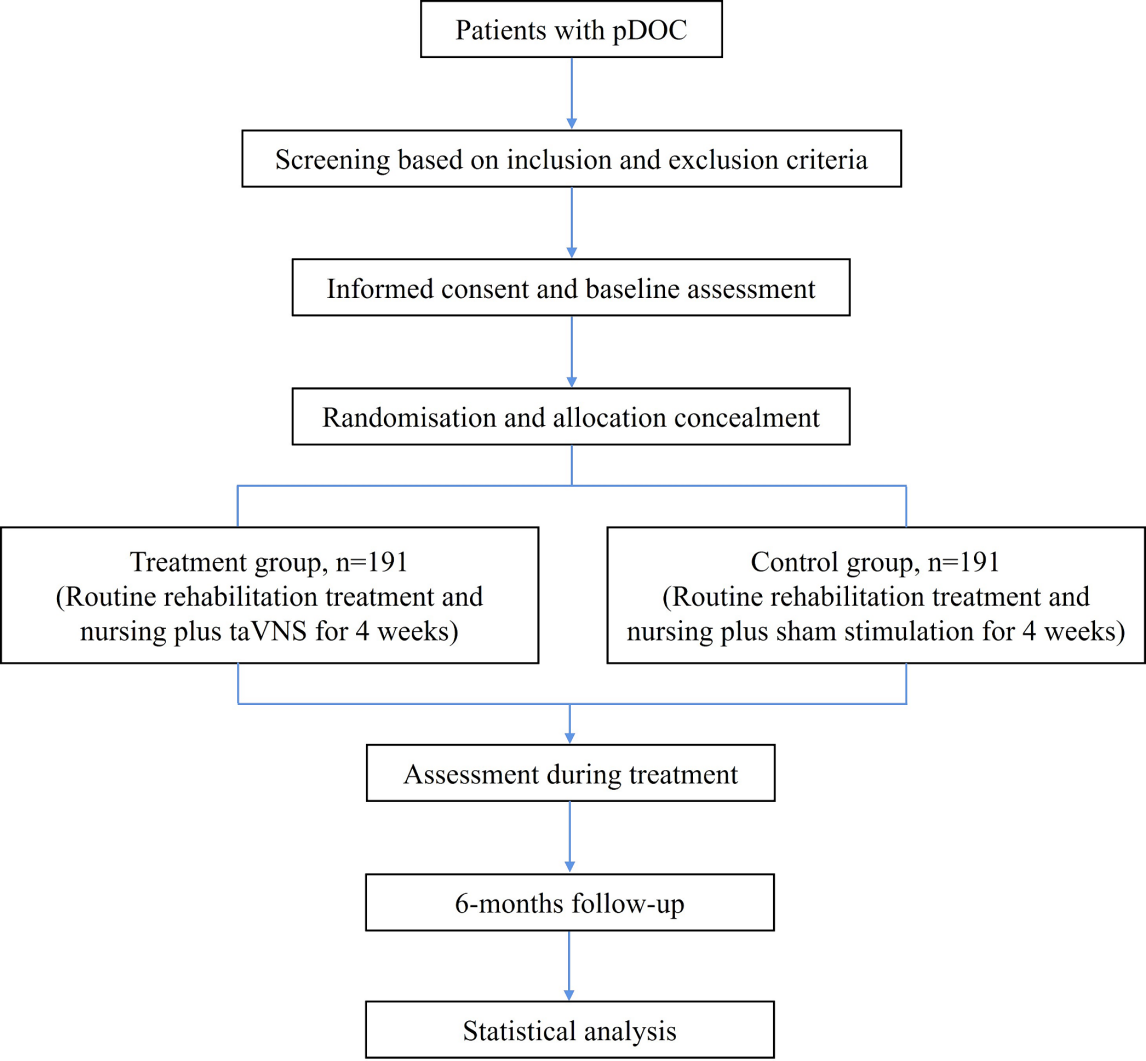
Study flowchart. pDOC, prolonged disorders of consciousness.

This is a prospective, multicenter, double-blind, stratified, two-arm RCT. This study protocol is designed according to the Declaration of Helsinki. It has been approved by the Ethics Committee of the Affiliated Rehabilitation Hospital of Nanchang University (SFYYXLL-PJ-2023-KY015) and has been registered at the Chinese Clinical Trial Registry (ChiCTR2400081978, https://www.chictr.org.cn/showproj.html?proj=221851). As the participants are patients with DOC, informed consent will be obtained from their legal representatives. The study design and final report will follow the Consolidated Standards of Reporting Trials (CONSORT) statement and its extension to non-pharmacologic treatment interventions. Figure 1 shows the study flowchart.

### Population

The study participants are patients with pDOC who will be recruited from 8 large and experienced centers of rehabilitation medicine: (1) Affiliated Rehabilitation Hospital of Nanchang University; (2) the First Affiliated Hospital of Nanchang University; (3) Ganzhou People’s Hospital; (4) the First Affiliated Hospital of Gannan Medical College; (5) Fuzhou First People’s Hospital; (6) Xinyu People’s Hospital; (7) Jiujiang First People’s Hospital; (8) Nanchang Hongdu Hospital of Traditional Chinese Medicine. Patients will be screened by trained medical personnel based on the inclusion and exclusion criteria.

Inclusion criteria: (1) Aged 18 to 65 years old; (2) Acquired brain injury patients with clear etiology; (3) Diagnosed as VS/UWS or MCS (based on 5 consecutive days of CRS-R evaluation, performed by two individuals, and consulted with a third party in case of dispute); (4) Fall in DOC up to 28 days; (5) The skin at the site of stimulation is intact; (6) Sign informed consent.

Exclusion criteria: (1) Patients whose vital signs are unstable; (2) Patients with active intracranial hypertension; (3) Patients with pacemakers, cochlear implants, or metal implants in the brain; (4) Patients with a history of cerebral nervous system disease prior to brain injury; (5) Patients with deep sedation caused by general anesthesia (e.g., propofol) or central sedatives (e.g., benzodiazepines, opioids); (6) Patients with bradycardia, atrial fibrillation, or atrioventricular block; (7) Pregnant patients.

Withdrawal criteria: (1) Recurrent seizures are difficult to control during treatment; (2) Life-threatening diseases (such as severe intracranial infections and cerebral hernia) occur; (3) Patients who consistently exhibit signs of pain below the threshold of given stimulus intensity; (4) Proactively exit.

### Sample size

The required sample size was calculated based on the results of our previous single-center RCT (24). In which, the difference of total CRS-R score between the taVNS group and the sham stimulation group after treatment was 10.93 ± 4.99 vs. 9.28 ± 4.38. We set the test power (1-β) to be 90%, the type I error rate (α) to be 0.05, and the group allocation of the two groups to be equal. The calculated sample size was N1 = N2 = 172. Considering that 10% of patients will be lost during follow-up, a total of 382 patients with pDOC will eventually be enrolled.

### Randomization and allocation concealment

The grouping scheme adopts stratified block randomization. Specifically, patients are first stratified according to the research center and then stratified according to the degree of DOC (VS/UWS or MCS). Then, patients are randomized 1:1 in variable block sizes, with stratification balancing by research center and degree of DOC. The randomization procedure is performed by independent statistical experts who are not involved in the study’s implementation or statistics. The study secretary places the generated random numbers and the grouping outcomes separately into opaque envelopes and sends them to each participating sub-center. After patients are confirmed to be enrolled, the sub-center doctors sequentially open the numbered envelopes to complete the grouping.

### Intervention protocol

**Figure 2:**
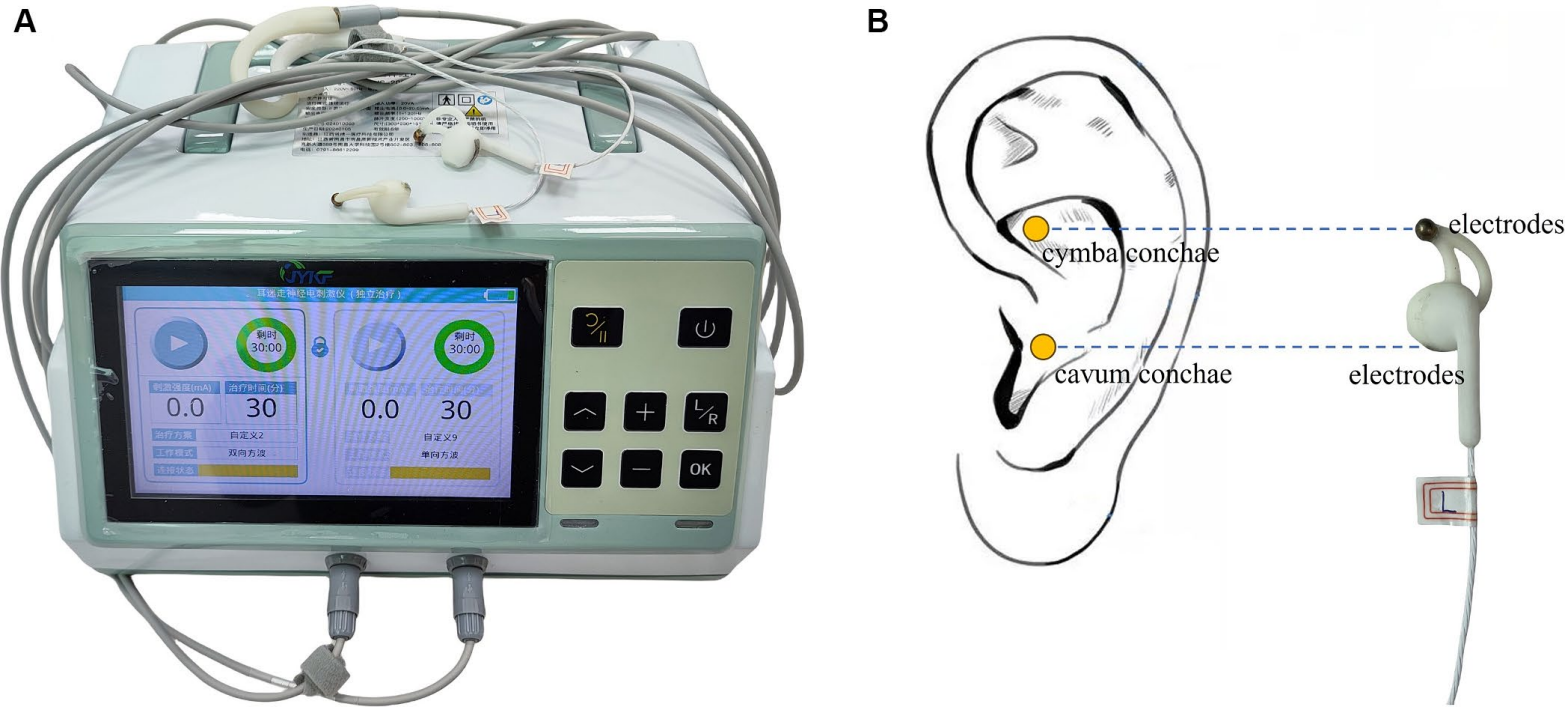
**(A)** Bilateral synchronous taVNS device; **(B)** stimulation sites and electrodes.

After grouping, both groups will undergo identical routine rehabilitation therapy and nursing. The active stimulation group will receive bilateral synchronized taVNS treatment (JY-VNS-200, Jingyi Medical Technology Co., Ltd., Jiangxi, China, Figure 2A). Electrotherapy is performed through a pair of metal electrodes, which are placed on the headphone-like stimulating end. The metal electrodes correspond directly to the cymba conchae and the cavum conchae (Figure 2B). Before treatment, the stimulation sites are thoroughly cleansed with alcohol to minimize impedance and ensure optimal conductivity. Treatment parameters: sine wave, 200 μs pulse width, 20 Hz frequency, 2 mA initial current intensity, 30 s on/30 s off cycle. The stimulation intensity will be gradually adjusted downwards in steps of 0.5 mA based on the patient’s tolerance (pain perception). To accurately distinguish pain from non-pain, the Nociception Coma Scale-Revised (NCS-R) will be utilized both initially and throughout the stimulation process, with a threshold of 4 points (25, 26). If the NCS-R score indicates pain (i.e., a score of ≥4), the stimulation intensity will be promptly reduced by 0.5 mA, and the NCS-R evaluation will be repeated. Patients who continue to exhibit signs of pain below the 0.5 mA threshold will be excluded from the study. To guarantee optimal contact between the electrodes and the ear skin, as well as to minimize the risk of electric burns, the device incorporates both an alarm function and a protection mechanism. These safety features will be activated whenever the electrodes fail to maintain adequate contact with the ear skin, such as when impedance exceeds 10 K Ω or the single pulse energy surpasses 8 mJ.

The device is presented with Mode A and Mode B. One is active stimulation, and the other is sham stimulation with no current output. Both modes exhibit identical screen displays and button operations (the current intensity can be adjusted, while other parameters are fixed to the values mentioned above). This makes researchers and device operators unaware of which stimulation is the active one. The two groups will receive treatment for 30 min per session, twice a day, 6 days a week for 4 weeks. The above treatment parameters and time refer to our previous single-center study (24) and peer studies (19, 23, 25). Bilateral synchronous taVNS will be performed at the same time before the start of the routine rehabilitation therapy in the morning and afternoon every day, under the operator’s continuous monitoring.

### Blinding and unblinding

The study is a double-blind design. The A/B Mode of the device effectively blinds the researchers. The participants are patients with pDOC who remain unaware of the study’s specifics. Furthermore, the evaluators and data analysts are kept blind to the grouping of patients. After the trial, the person in charge of blinding who did not participate in the study will break the blinding. When patients experience serious complications (such as cardiac arrest) during the trial and they are suspected to be related to taVNS, the sub-center can call the person in charge of blinding for emergency unblinding.

### Data collection

After enrollment, demographics and baseline data of patients in both groups will be collected, including gender, age, cause of injury, duration of DOC, CT results (subarachnoid hemorrhage, hydrocephalus), pupillary light reflex (none, one side, both sides), cranial surgery (with or without), tracheotomy (with or without), multiple injuries (with or without), initial CRS-R, Full Outline of Unresponsiveness (FOUR), and Glasgow Coma Scale (GCS) scores.

**Figure 3:**
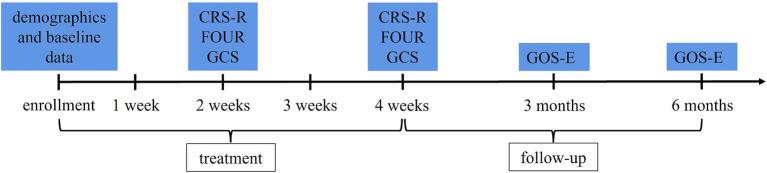
Evaluation process. CRS-R, Coma Recovery Scale-Revised; FOUR, Full Outline of Unresponsiveness; GCS, Glasgow Coma Scale; GOS-E, Extended Glasgow Outcome Scale.

Patients in both groups will be evaluated via CRS-R, FOUR, and GCS scores after 2 weeks of treatment and at the end of treatment (after 4 weeks of treatment). The improvement in the CRS-R scores at the end of treatment is the primary outcome. Extended Glasgow Outcome Scale (GOS-E) scores will be followed up at 3 and 6 months after the end of treatment. During follow-up, if the patients are still in the hospital, they will be evaluated in the ward. If the patients are discharged, they will be evaluated via structured phone interviews with themselves, their family members, or caregivers. According to reports, the assessment of GOS-E via phone is a valid alternative to face-to-face interviews when face-to-face contact is not possible. The level of agreement (Cohen’s weighted κ) between the two is good (27). The evaluation process during treatment and follow-up is shown in Figure 3. The total length of hospital stay and mortality during treatment and follow-up will also be recorded.

The cardiovascular system is doubly innervated by the sympathetic and parasympathetic nerves. The vagus nerve is a mixed cranial nerve that contains parasympathetic nerve fibers. In theory, stimulating the vagus nerve may increase the risk of its mediated bradycardia and hypotension. Heart rate variability (HRV) analysis can provide information on the balance between the sympathetic and parasympathetic pathways (28). It is a useful tool for evaluating cardiac autonomic regulation (28). Therefore, we will use a heart rate chest strap (Maijin Intelligent Technology Co., Ltd., Qingdao, China) and the Elite HRV smartphone application (Elite HRV Inc., Asheville, NC, United States) to collect and analyze patients’ HRV information. Specifically, placing the sensor of the chest strap at the level of the heart in front of the chest. Adjusting the length of the elastic strap to ensure close contact between the electrode area and the skin. Connecting the chest strap to the Elite HRV application on the phone via Bluetooth. We will collect and analyze HRV information of the patients for 30 min before and during the first taVNS, including mean heart rate, mean RR, the standard deviation of normal-to-normal RR intervals (SDNN), the root mean square of successive differences (RMSSD), the proportion of NN50 divided by the total number of normal-to-normal RR intervals (PNN50), low-frequency (LF) power, high-frequency (HF) power, total power, and LF/HF ratio. The chest strap records the cardiac electrical activity and extracts heart rate data from the electrocardiogram (ECG) waveforms, which has detection results comparable to Holter ECG (29–31). For blood pressure, we will record it once separately before and during the patient’s first taVNS. In addition to our recordings, ECG monitoring will also be performed on patients during each stimulation to detect vital signs.

Poor skin and electrode contact may lead to skin burns. Although the device provides burn risk alarm and protection mechanisms, we will still record skin burns at the stimulation sites, including exudation, blisters, and other skin damage. We will record any adverse events that may occur in various systems during the trial period, which may affect the patient’s prognosis. These include epilepsy, paroxysmal sympathetic hyperactivity, hydrocephalus, intracranial infection (nervous system); deep vein thrombosis, pulmonary embolism, cardiac arrest (cardiovascular system); pulmonary infection, acute respiratory distress syndrome (respiratory system); gastric bleeding (digestive system); and urinary tract infection (urinary system). These adverse events are largely unrelated to taVNS. In addition, we will collect records on the type and volume of routine rehabilitation therapy for both groups during the trial period.

### Data management

The data collected from each patient will be recorded in a standardized case report form (CRF), and the CRF will be transmitted to the coordinating center (Affiliated Rehabilitation Hospital of Nanchang University) after the trial is completed. The researchers responsible for data management at the center will store these data anonymously on the Research Electronic Data Capture platform. Only researchers responsible for data management can access its content. After all data are stored, the database will be locked and sent to the research statistician for analysis according to the predetermined statistical analysis plan.

### Statistical analysis

Statistical analysis will be performed using R software. For demographic and baseline data, the quantitative data that conform to a normal distribution will be expressed as mean ± standard deviation ( $\bar{x}$ ± *s*) and differences between groups will be analyzed using the independent samples *t*-test. The quantitative data that do not conform to a normal distribution will be expressed as median (interquartile range, IQR) and differences between groups will be analyzed using the Mann–Whitney U test. The qualitative data will be analyzed using the chi-square test or Fisher’s exact test. For the efficacy indicators, in order to detect changes in CRS-R, FOUR and GCS scores over time and differences between groups, a linear mixed-effects model (LMM) with repeated-measures analysis from the “nlme” package in R software will be used. *Post-hoc* exploratory subgroup analyses will explore the effects of taVNS on subgroups according to different levels of consciousness and different etiologies. For safety indicators, HRV and blood pressure data were tested for normal distribution and analyzed using the paired-samples *t*-test or Wilcoxon Signed-rank test. Differences between groups in the incidence of adverse events were analyzed using the chi-square test or Fisher’s exact test. *p* < 0.05 was considered statistically significant.

## Discussion

Despite great efforts in medication therapy, neuromodulation and physical rehabilitation, the successful treatment strategy of DOC remains limited, primarily due to a profound lack of comprehension of the underlying pathophysiology (32). In recent years, there has been a growing interest in VNS as a potential therapeutic approach, encompassing both invasive and non-invasive techniques. The invasive method involves surgically transmitting electrical pulses directly to the exposed cervical vagus nerve. Notably, the vagus nerve asymmetrically innervates the heart, and stimulating the right cervical vagus nerve can result in electrical signals being directly fed into the sinoatrial node, thereby heightening the risk of adverse cardiac events, such as arrhythmia. Conversely, stimulating the left vagus nerve poses a significantly lower risk of such complications. Thus the left vagus nerve is typically the preferred target for invasive method (33). However, this method is expensive and complex to operate. TaVNS, on the other hand, offers an economical, easier to implement and noninvasive alternative. It stimulates the auricular branch of the vagus nerve (ABVN) by targeting the skin of the outer ear, specifically the cymba conchae and cavum conchae (34).

Our previous study demonstrated the efficacy and safety of taVNS through the left ear. It provided the highest level of Class II evidence currently available (24). However, it was a single-center study with a limited sample size. Additionally, as a preliminary study, taVNS was solely administered through the left ear for safety considerations. Nevertheless, some literature indicated that non-invasive stimulation of the ABVN was not associated with adverse cardiac events. This is attributed to its selective stimulation of afferent fibers, which are processed by the brain before reaching the heart, rather than directly activating the heart’s pacemaking nodes (35). This effectively mitigates the side effects associated with efferent (visceral) fiber activation. Additionally, several studies have corroborated the security of bilateral taVNS (36, 37). Thereby, our current protocol aims to administer bilateral synchronized taVNS, aiming to boost treatment effectiveness, taking into account that brain damage can occur on the left, right, or both sides in DOC patients.

The ABVN is the only branch of the vagus nerve on the surface of the body, which mainly distributed in the external auditory meatus and concha (cymba conchae and cavum conchae, the cymba conchae is innervated exclusively by the ABVN) (38). The latter is usually considered as the ideal target area for taVNS. The ABVN transmits stimuli from the concha to the spinal trigeminal nucleus and the solitary tract nucleus (39), which is then projected and extended to the cerebral cortex and subcortical regions related to consciousness control (34). Neuroelectrophysiology and neuroimaging play pivotal roles in elucidating the mechanisms by which taVNS affects brain function in patients with DOC. The combination study of taVNS and electroencephalogram (EEG) found that taVNS improved the consciousness level of patients with MCS by enhancing the high-frequency relative power spectrum energy and functional connectivity (FC) of the frontal and parietal lobes (40). In a longitudinal case study (41), the EEG power in the alpha band gradually increased, potentially indicating neural network integration and cortical activity enhancement. Furthermore, a study combining arterial spin labeling-functional magnetic resonance imaging (ASL-fMRI) discovered that preserved auditory function may serve as a prerequisite for taVNS responders among patients with DOC. Additionally, taVNS may activate the salient network, limbic system, and interoceptive system to improve the condition of these patients (42). Yu et al. (19) indicated that TaVNS increased the FC between posterior cingulate/precuneus and hypothalamus, thalamus, ventral medial prefrontal cortex, superior temporal gyrus. Drawing from numerous research findings, Briand et al. (39) proposed a vagal cortical pathways model. They further outlined six possible mechanisms by which taVNS promotes consciousness recovery. In addition, in molecular mechanism research, VNS showed potential for DOC by reducing cell apoptosis, regulating neurotransmitters, decreasing inflammatory responses, and lowering blood–brain barrier permeability (34). However, the exact mechanism is still not fully understood. Further validation research is necessary, as the exact mechanism can provide information for developing more targeted and effective treatment strategies.

In taVNS studies for other diseases, the current intensity was typically determined based on the patient’s perceptual threshold [e.g., 200% of the perceptual threshold (43)] or pain threshold [strongest painless stimulus (44)]. However, these methods are not applicable for patients with DOC due to their perception and communication deficits. NCS-R is a validated and highly sensitive tool for assessing the nociceptive pain responses of patients with DOC through motor, verbal and facial aspects (26). In this protocol, we use NCS-R to assess the tolerance of patients to taVNS. Specifically, the current intensity is gradually reduced in steps of 0.5 until the patients exhibit no pain response (NCS-R score < 4).

Although reports indicating that bilateral taVNS has little impact on parasympathetic nerves and is considered safe (36, 37), we will still evaluate its effects on heart rate and blood pressure. HRV serves as a crucial metric for assessing cardiac autonomic regulation by quantifying variations in sinus rhythm (28). Therefore, we will use HRV to measure the impact of taVNS on the sympathetic/parasympathetic balance. Typically, HRV is calculated using a Holter ECG, a process that can be quite intricate. Given that this is a multicenter study, the tools and methods for collecting and analyzing Holter ECG data vary significantly across sub-centers. Additionally, large-sample multicenter trials require procedures that are straightforward and easily executable. Hence, we opt for a solution involving a chest strap coupled with the EliteHRV software. This solution provides acceptable agreement compared to ECG (30, 31). In our protocol, this alternative ensures consistency, simplicity, and operability of multi-center trials.

This protocol still exists some limitations. Firstly, given the complexities of neuroimaging and electrophysiology in multicenter trials, our focus will primarily be on patients’ behavioral outcomes, while neglecting neuroimaging or electrophysiology programs. Secondly, due to the difficulties associated with continuously collecting blood pressure data, we will limit our collection to a single measurement before and during taVNS. This approach may somewhat diminish the statistical power compared to continuous blood pressure monitoring. Thirdly, the parameters utilized in this protocol are based on our previous single-center RCT and other relevant studies. There is currently no consensus on the optimal parameters for taVNS. Future studies should focus on determining the optimal parameters for specific patient populations, as well as investigating potential dose–response relationships and individual factors that may affect treatment outcomes.

In conclusion, the clinical treatment of DOC is challenging. TaVNS is an economical, non-invasive, promising, bottom-up neuromodulation. This protocol aims to provide multicenter, large-sample, and more effective Class II evidence for the efficacy and safety of taVNS for DOC. It will advance the field of treatment options for patients with DOC.

## Ethics statement

The studies involving humans were approved by the Ethics Committee of the Affiliated Rehabilitation Hospital of Nanchang University. The studies were conducted in accordance with the local legislation and institutional requirements. The participants provided their written informed consent to participate in this study.

## Author contributions

YW: Conceptualization, Writing – original draft. LY: Resources, Writing – review & editing. WL: Resources, Writing – review & editing. QZ: Investigation, Writing – review & editing. MH: Visualization, Writing – review & editing. LZ: Supervision, Writing – review & editing. ZF: Conceptualization, Methodology, Writing – review & editing. YB: Conceptualization, Project administration, Writing – review & editing.
